# Spatiotemporal patterns of influenza incidence changes in mainland China, 2004–2020

**DOI:** 10.3389/fpubh.2025.1667299

**Published:** 2025-10-27

**Authors:** Shengcong Tao, Yirong Guo

**Affiliations:** ^1^Health Statistics Information Center, Lanzhou, China; ^2^Longdong University, Qingyang, China

**Keywords:** influenza, temporal trend, spatial pattern, heterogeneity, China

## Abstract

**Background:**

Influenza incidence varies significantly over time and across regions. However, the current understanding of the spatiotemporal patterns of influenza incidence changes in mainland China is limited, which significantly hampers the relevant authorities' ability to prevent and control influenza outbreaks.

**Objectives:**

This study aims to update and enhance the understanding of influenza incidence patterns in China through a large-scale spatial and temporal analysis of cases among a population of ~1.4 billion, providing a reference for planning and optimizing prevention and treatment strategies.

**Methods:**

Monthly surveillance data on influenza incidence across all provinces of mainland China from 2004 to 2020 were collected, and the intra-annual distribution characteristics were statistically summarized. An exponential trend model was used to estimate inter-annual trends on monthly, quarterly, and annual scales for each province, and their spatial patterns were explored and analyzed. The spatial heterogeneity was examined using linear regression models.

**Results:**

The average annual incidence of influenza in mainland China from 2004 to 2020 was 31.57 cases per 100,000 people, with significant outbreaks occurring in 2009 and 2019, exceeding the expected values by 142% and 323%, respectively. It reveals clear regional differences, with a higher prevalence in the east and a lower prevalence in the west, indicating localized clustering. The intra-annual distribution pattern follows a U-shaped curve, characterized by a high incidence in winter and a low incidence in summer. The monthly, seasonal, and annual influenza incidence showed an overall increasing trend, with a more pronounced rise after 2010, particularly reflected in the increased number of cases during winter months. The trend varies by geographic location, showing a significant negative correlation with elevation and a significant positive correlation with longitude. Provinces with larger populations experience greater fluctuations and faster growth.

**Conclusions:**

The influenza prevention and control situation in mainland China remains severe. Macro-level strategies should account for these temporal and spatial patterns, allocate limited medical resources effectively, and respond actively and promptly to potential outbreaks in key regions and at critical times.

## 1 Introduction

Influenza and its complications pose a continuing threat to global public health (1, 2). It is an acute respiratory infectious disease caused by the influenza virus that spreads worldwide, with main symptoms including fever, cough, headache, sore throat, muscle pain, nasal congestion, runny nose, and sneezing. Most infected individuals recover from fever and other symptoms within a few weeks without requiring hospitalization. However, influenza can lead to severe complications and even the risk of death in high-risk groups, including infants and young children, the older adults, pregnant women, and patients with underlying cardiopulmonary conditions (3, 4). The influenza virus is prone to mutations, highly contagious, and spreads through various transmission routes (4), leading to widespread susceptibility in the population, a high incidence, and a broad impact, resulting in cluster outbreaks or pandemics. It is estimated that about 5%−10% of adults and 20%−30% of children globally contract influenza each year, resulting in 3–5 million severe cases and 290,000 to 650,000 deaths (5–11). The outbreak of influenza disrupts the regular order of life, work, and education, impedes stable socioeconomic development, and imposes a substantial disease burden on countries worldwide (1, 3, 12–14). The risk of future pandemics remains (9), as ongoing viral mutations could lead to the emergence of new influenza strains (15). Therefore, exploring the patterns of influenza activity is crucial for responding to impending influenza outbreaks (16–18).

Influenza is a preventable respiratory infectious disease, and clarifying its activity patterns is crucial for effective outbreak management, helping to avoid unnecessary economic losses and casualties (8, 19). Researchers worldwide have comprehensively investigated influenza dynamics through multifaceted approaches encompassing epidemiological analyses (20), time-series modeling (21, 22), and predictive forecasting (23). Influenza activity exhibits distinct seasonal and regional patterns (9) as it is influenced by a variety of factors such as weather conditions, socioeconomic factors and demographic characteristics (9, 19, 24–29). Due to the complex, non-linear relationship between environmental conditions and influenza incidence (19, 30), influenza epidemics exhibit significant spatiotemporal heterogeneity (19, 31–34). Consequently, each influenza pandemic exhibits distinctive characteristics in viral subtypes, affected populations, geographic distribution, and mortality rates. However, most current studies focus on single-dimensional analyses, and the exploration of spatiotemporal heterogeneity remains in an active stage of development.

Exploring the spatiotemporal patterns of influenza incidence change in different regions is crucial for developing effective prevention, control, and management measures (19). Particularly, analyzing long-term changes in influenza incidence across large populations and expansive geographic regions will help uncover the underlying patterns of influenza dynamics (8) and lay a critical foundation for subsequent prevention and control efforts (35). China is a geographically, economically, and climatologically diverse country with a population of 1.4 billion and a heavy disease burden of influenza (36). Due to its vast territory, complex climate, and diverse environments, influenza incidence exhibits marked heterogeneity (9, 36–41). Despite significant improvements in influenza prevention and control with the continuous advancement of healthcare services, the large population base and complex pathogenic factors pose substantial challenges (19), resulting in a heavy disease burden (9, 36, 42). While prior research has explored the patterns of influenza activity in China, yielding substantial and valuable insights (36, 40, 43–46), most studies have been limited to single-dimensional variations or specific regions, neglecting the association between static and dynamic characteristics (35, 45, 47–51). Owing to differences in study periods, regions, and objectives, the current understanding of influenza activity patterns is often inconsistent or incomplete (9). Furthermore, a comprehensive knowledge of nationwide patterns remains insufficient (19), and certain existing findings have become outdated (8), creating substantial challenges for influenza prevention and control efforts. In recent years, China has witnessed an upward trend in influenza incidence (42), with certain regions experiencing repeated outbreaks (19, 52). Despite its annual occurrence, the timing and scale of outbreaks vary considerably. Thus, analyzing the spatiotemporal patterns of influenza incidence is crucial for effective epidemic surveillance and response (8).

However, detailed and comprehensive spatiotemporal analyses of influenza incidence in China remain scarce (19), particularly those integrating data from multiple provinces. In light of this gap, we conducted a large-scale spatiotemporal analysis based on monthly influenza surveillance data. This study has three objectives: (i) to investigate the within-year distribution and interannual variability of influenza incidence in mainland China from 2004 to 2020; (ii) to identify the spatial patterns of influenza incidence characteristics;(iii) to reveal the associations among these characteristics. The findings are expected to update and refine the understanding of current patterns in influenza incidence across mainland China, providing a scientific basis for the control and prevention of influenza.

## 2 Materials and methods

### 2.1 Data source

The influenza incidence is a unique and valuable indicator for measuring regional influenza activity. The influenza incidence data used in this study are publicly available from the China Public Health Sciences Data Center of the Chinese Center for Disease Control and Prevention (https://www.phsciencedata.cn). We obtained monthly influenza incidence data for 31 provinces in mainland China from 2004 to 2020. This dataset originates from the National Notifiable Infectious Diseases Reporting Information System (NIDRIS), in which influenza is one of the statutorily reportable infectious diseases. The NIDRIS is a key part of China's public health system, involving over 70,000 medical and health institutions nationwide. It mandates all levels and types of medical and health institutions to report influenza cases in a standardized format, aiming to provide timely and accurate data to support public health decisions and to effectively prevent and control the spread and outbreaks of infectious diseases. When a patient presents with a fever (body temperature ≥38 °C) along with either a cough or a sore throat, the initial consulting doctor must report it through this system within 24 h. Due to challenges in data collection, data from Hong Kong, Macao, and Taiwan are not included. The public dataset is aggregated by month and province and does not distinguish between influenza-like illness (ILI) and laboratory-confirmed reports, nor does it include case-level details. Therefore, it contains no personal information or confidentiality concerns. Additionally, since the dataset is publicly accessible, no ethics committee approval is required.

The boundaries of China's administrative districts are from the National Catalog Service for Geographic Information (https://www.webmap.cn). Additional data were obtained from the National Basic Science Data Center (https://www.nbsdc.cn).

### 2.2 Statistical analysis

The subject of the statistical analysis is the monthly influenza incidence in each province, which is defined as the number of new cases per month due to influenza infection, in units of per 10,0000 people. We used the natural exponential model to estimate change trends on different temporal scales. Its formula is shown below:


$$\begin{array}{l} IIR\left(iYear\right)=a\cdot e^{b\times iYear} \end{array}$$

Where *IIR* indicates the influenza incidence rate and *iYear* denotes the yearly ordinal number. Starting from 2003, the corresponding iYear for 2004 is 1, for 2005 is 2, and so on. *a* and *b* are the parameters of the equations estimated by the least squares method. The coefficient of determination (*R*^2^) was used to assess the goodness of fitting, and the F-test was employed to evaluate the significance level of the change trend (*p* < 0.05). This trend model describes a non-linear relationship, indicating that *IIR* exhibits an exponential growth or decay trend relative to *iYear*. When *b* > 0, the trend is increasing; when *b* < 0, the trend is decreasing.

We conducted a spatial analysis to compare influenza incidence across provinces in mainland China. To facilitate comparison, we categorized the change characteristics and assigned them distinct colors. These analyses were performed using ArcMap software (version 10.6). Additionally, we employed the Pearson correlation coefficient (*r*) and linear regression models to investigate the relationships between the characteristics and geographical factors, including longitude, latitude, and elevation. The strength of these associations was measured by *R*^2^ and Root Mean Square Error (RMSE).

## 3 Results

### 3.1 Temporal characteristics of influenza incidence

#### 3.1.1 Intra-annual distribution

**Figure 1:**
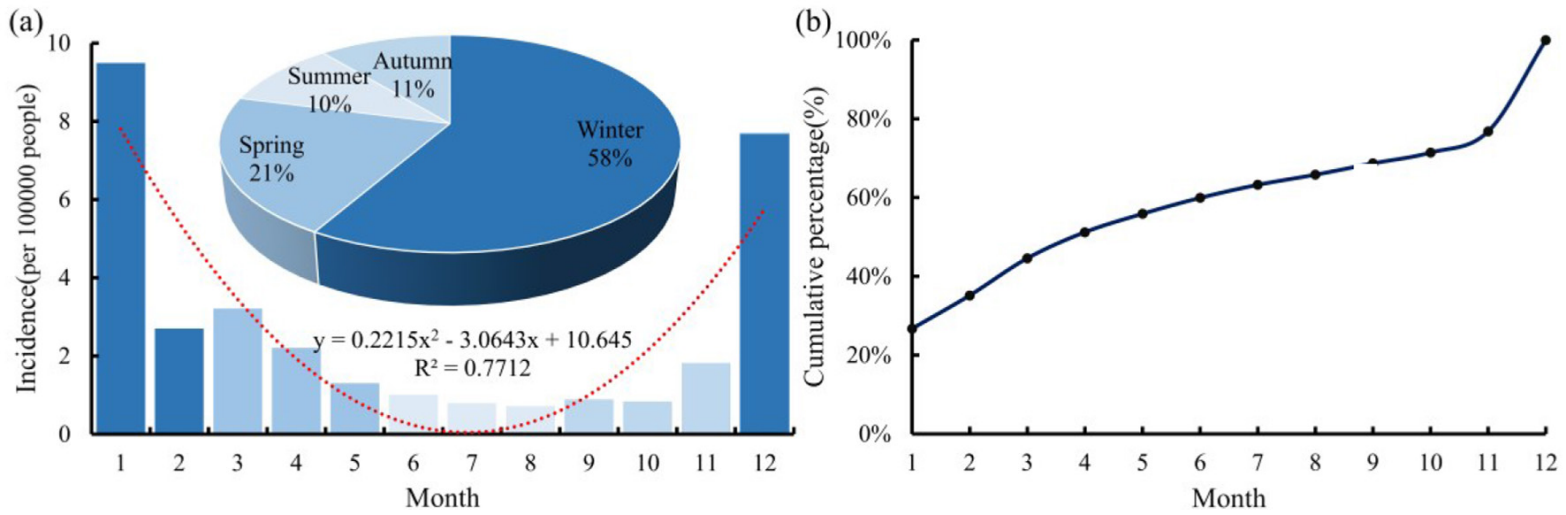
Average **(a)** and cumulative curve **(b)** of monthly influenza incidence in mainland China from 2004 to 2020.

Based on the monthly incidence data of 31 provinces in mainland China from 2004 to 2020, we statistically calculated the average value for each month and the percentage for each season (Figure 1). There were clear differences in influenza incidence between months, especially between December and November, as well as between January and February, with differences of 3.2 and 2.5 times, respectively. Influenza incidence is highest in January, followed by December, with these 2 months accounting for ~53% of the total annual cases. Starting in March, incidence decreases month by month, with only sporadic cases in July and August, followed by a gradual increase afterwards.

To visually illustrate the intra-annual distribution pattern, we fitted the multi-year averaged monthly influenza incidence and plotted the annual distribution curve. As shown in Figure 1a, the curve is an upward-opening parabola, with a trough in July and peaks in January and December, indicating a *U*-shaped variation on a monthly scale. The incidence is lowest in summer, accounting for about 10% of the annual total, followed by autumn, spring, and winter, which account for 11%, 21%, and 58%, respectively.

The cumulative curve of influenza incidence throughout the year is shown in Figure 1b. From January to April, the cumulative number of cases increased significantly, accounting for ~51% of the total cases for the year. Subsequently, the increasing trend became moderate, with only about 21% added from May to November. In December, there was another surge in cases. These results indicate a distinct seasonality of influenza incidence in mainland China, with outbreaks occurring during the winter months.

#### 3.1.2 Inter-annual change

**Figure 2:**
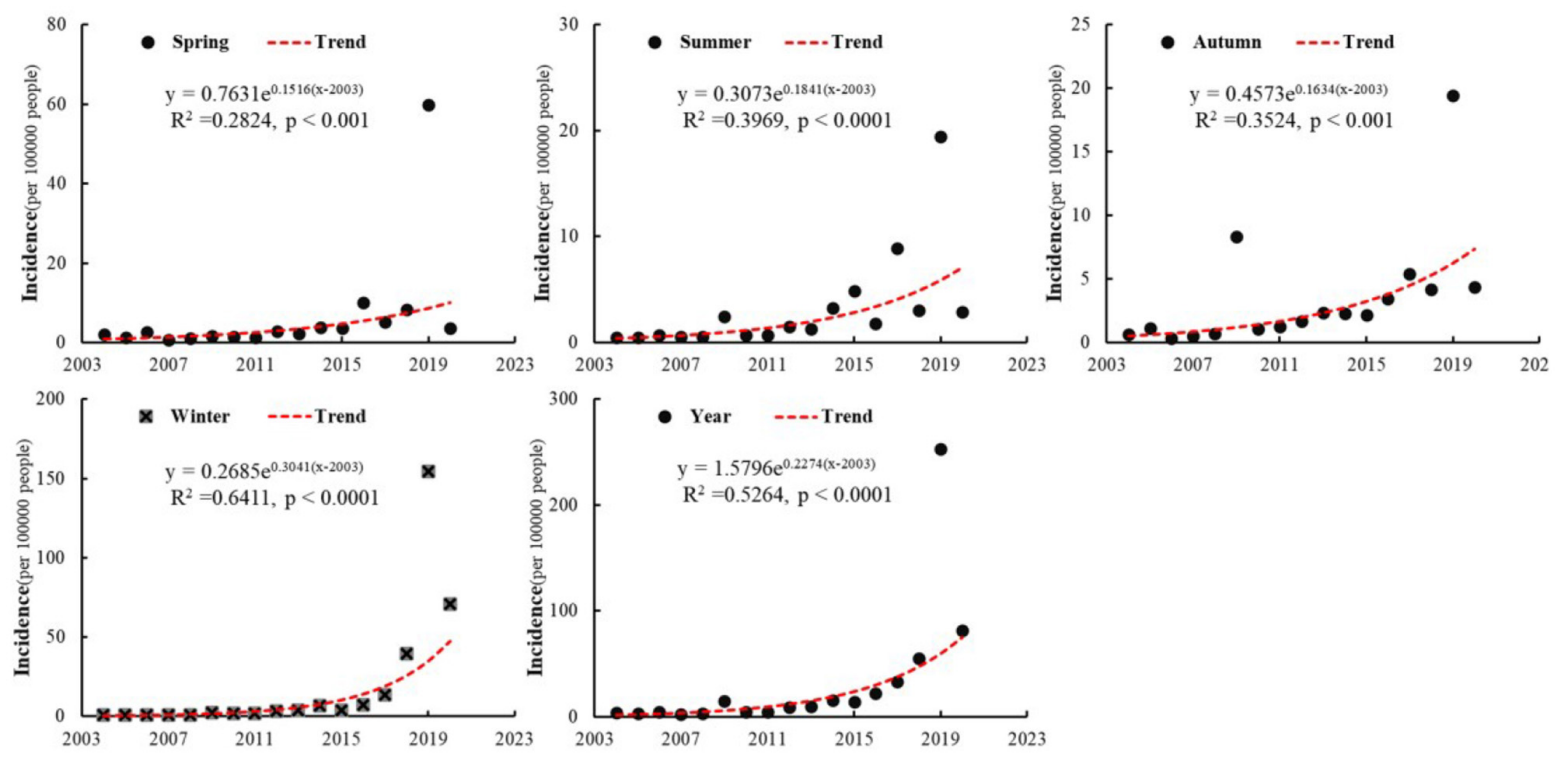
Trends of seasonal and annual influenza incidence in mainland China from 2004 to 2020.

To compare interannual variations, we plotted the trend of influenza incidence for each season by summarizing the complete monthly surveillance data from each province in mainland China (Figure 2). From 2004 to 2020, influenza incidence in all four seasons showed a significant increasing trend. The growth rates, listed in descending order, are winter, summer, autumn, and spring. Winter not only has the highest growth rate but also the most significant trend (*p* < 0.001, *R*^2^ = 0.64). The increase across the four seasons led to a rise in the annual incidence rate from 3.81 per 100,000 people in 2004 to 81.58 per 100,000 in 2020. After 2010, this growth trend became even more pronounced. The average annual influenza incidence from 2011 to 2020 was nine times higher than that from 2004 to 2010. Consequently, the study period can be divided into two phases based on annual incidence: a slow growth phase from 2004 to 2010 and a rapid growth phase from 2011 to 2020. Additionally, influenza outbreaks occurred in China in 2009 and 2019, with their annual incidence rates being more than three times those of the previous and following years, exceeding expected values by 142% and 323%, respectively.

Table 1 summarizes the change rates of monthly influenza incidence for each province in mainland China from 2004 to 2020, with statistically significant results marked in bold. The monthly influenza incidence in the 31 provinces of mainland China generally shows an increasing trend (>96%), with 80% of these provinces passing the significance test. Anhui, Beijing, Fujian, Guangdong, Hebei, Henan, Hubei, Hunan, Jiangxi, Liaoning, Shandong, Shanxi, and Zhejiang, a total of 13 provinces, show a significant increasing trend in the monthly incidence. Notably, the populations of these 13 provinces account for nearly half of China's total population. Four provinces, Guizhou, Sichuan, Tianjin, and Tibet, exhibited decreasing trends in sporadic months. Except for the significant trends in July and September in Tibet, the trends in the other provinces were not significant. The influenza incidences in January, February, and December across all provinces exhibit the most significant trends, with the highest rates of change. The trends in April, May, October, March, and June are less significant, with at least six provinces failing the significance test. In summary, from 2004 to 2020, the monthly incidence of influenza in mainland China exhibited an increasing trend, primarily during the winter months.

**Table 1:** Change rates of monthly influenza incidence across provinces in mainland China from 2004 to 2020.

**Province/Month**	**1**	**2**	**3**	**4**	**5**	**6**	**7**	**8**	**9**	**10**	**11**	**12**
Anhui	**0.40**	**0.35**	**0.29**	**0.24**	**0.26**	**0.27**	**0.28**	**0.19**	**0.20**	**0.23**	**0.25**	**0.35**
Beijing	**0.55**	**0.50**	**0.45**	**0.46**	**0.42**	**0.34**	**0.28**	**0.26**	**0.26**	**0.29**	**0.30**	**0.46**
Fujian	**0.42**	**0.33**	**0.21**	**0.16**	**0.22**	**0.22**	**0.21**	**0.27**	**0.26**	**0.28**	**0.36**	**0.39**
Gansu	**0.18**	**0.19**	**0.11**	0.07	0.08	**0.11**	**0.17**	**0.16**	**0.16**	**0.15**	**0.13**	**0.12**
Guangxi	**0.32**	**0.18**	0.10	0.11	**0.14**	**0.14**	**0.13**	**0.19**	**0.18**	**0.19**	**0.25**	**0.41**
Guangdong	**0.47**	**0.31**	**0.18**	**0.16**	**0.22**	**0.21**	**0.25**	**0.25**	**0.25**	**0.27**	**0.27**	**0.40**
Guizhou	0.16	0.15	**0.04**	**0.00**	**0.03**	**0.05**	0.10	**0.03**	**−0.01**	**−0.05**	**0.02**	**0.13**
Hainan	**0.42**	**0.37**	**0.25**	**0.17**	0.12	**0.19**	**0.24**	**0.24**	**0.19**	**0.24**	**0.28**	**0.39**
Hebei	**0.24**	**0.19**	**0.17**	**0.15**	**0.13**	**0.16**	**0.16**	**0.15**	**0.13**	**0.12**	**0.12**	**0.17**
Henan	**0.34**	**0.29**	**0.24**	**0.23**	**0.26**	**0.27**	**0.25**	**0.16**	**0.19**	**0.23**	**0.22**	**0.27**
Heilongjiang	**0.29**	**0.39**	**0.39**	**0.34**	**0.22**	**0.18**	**0.17**	**0.15**	**0.16**	**0.17**	0.13	**0.29**
Hubei	**0.49**	**0.30**	**0.31**	**0.29**	**0.28**	**0.26**	**0.25**	**0.18**	**0.16**	**0.25**	**0.29**	**0.39**
Hunan	**0.42**	**0.31**	**0.20**	**0.23**	**0.28**	**0.26**	**0.26**	**0.25**	**0.25**	**0.27**	**0.28**	**0.40**
Jilin	**0.35**	**0.32**	**0.35**	**0.26**	**0.22**	0.10	0.05	0.03	0.07	0.09	0.03	**0.18**
Jiangsu	**0.35**	**0.32**	**0.25**	**0.17**	**0.15**	**0.23**	**0.18**	**0.20**	0.07	**0.26**	**0.27**	**0.26**
Jiangxi	**0.35**	**0.26**	**0.16**	**0.13**	**0.14**	**0.15**	**0.14**	**0.20**	**0.17**	**0.21**	**0.19**	**0.26**
Liaoning	**0.30**	**0.33**	**0.30**	**0.27**	**0.23**	**0.29**	**0.36**	**0.24**	**0.25**	**0.26**	**0.32**	**0.41**
Neimenggu	**0.39**	**0.36**	**0.32**	0.17	0.02	**0.23**	**0.28**	**0.29**	0.19	**0.26**	**0.25**	**0.26**
Ningxia	**0.19**	**0.14**	0.05	0.04	0.03	0.05	0.09	**0.14**	0.11	0.03	0.02	0.12
Qinghai	**0.22**	**0.18**	0.06	0.09	**0.13**	**0.20**	**0.15**	**0.13**	**0.19**	**0.10**	**0.21**	**0.29**
Shandong	**0.39**	**0.39**	**0.36**	**0.36**	**0.38**	**0.35**	**0.38**	**0.29**	**0.29**	**0.34**	**0.30**	**0.42**
Shanxi	**0.40**	**0.43**	**0.42**	**0.33**	**0.28**	**0.31**	**0.34**	**0.33**	**0.29**	**0.40**	**0.31**	**0.35**
Shaanxi	**0.43**	**0.32**	**0.31**	**0.25**	**0.25**	**0.24**	**0.27**	**0.28**	0.15	**0.24**	**0.25**	**0.33**
Shanghai	**0.44**	**0.35**	**0.25**	**0.28**	0.20	**0.19**	0.16	0.03	**0.16**	**0.17**	**0.23**	**0.35**
Sichuan	**0.22**	**0.17**	0.09	0.00	0.04	0.04	0.05	0.05	0.05	0.12	0.12	**0.18**
Tianjin	**0.25**	**0.23**	**0.19**	0.05	0.03	0.14	0.09	0.04	−0.06	0.11	**0.20**	**0.22**
Tibet	0.12	−0.01	−0.21	−0.08	0.01	−0.08	–**0.09**	−0.08	–**0.17**	0.00	−0.20	0.04
Xinjiang	**0.28**	**0.26**	0.22	0.19	**0.06**	**0.13**	**0.19**	**0.21**	0.17	0.15	**0.21**	**0.26**
Yunnan	**0.31**	**0.25**	0.12	0.10	**0.17**	**0.21**	**0.22**	**0.23**	0.06	0.15	**0.21**	**0.35**
Zhejiang	**0.41**	**0.31**	**0.17**	**0.13**	**0.22**	**0.19**	**0.18**	**0.15**	**0.16**	**0.24**	**0.28**	**0.39**
Chongqing	**0.29**	**0.15**	0.07	0.04	0.08	**0.10**	**0.09**	**0.10**	0.07	0.14	**0.17**	**0.26**
Nation	**0.33**	**0.25**	**0.16**	**0.13**	**0.17**	**0.18**	**0.20**	**0.18**	**0.14**	**0.16**	**0.18**	**0.27**

Change rates that pass the F-test are bolded.

### 3.2 Spatial patterns of influenza incidence change

#### 3.2.1 Spatial distribution of influenza incidence

**Figure 3:**
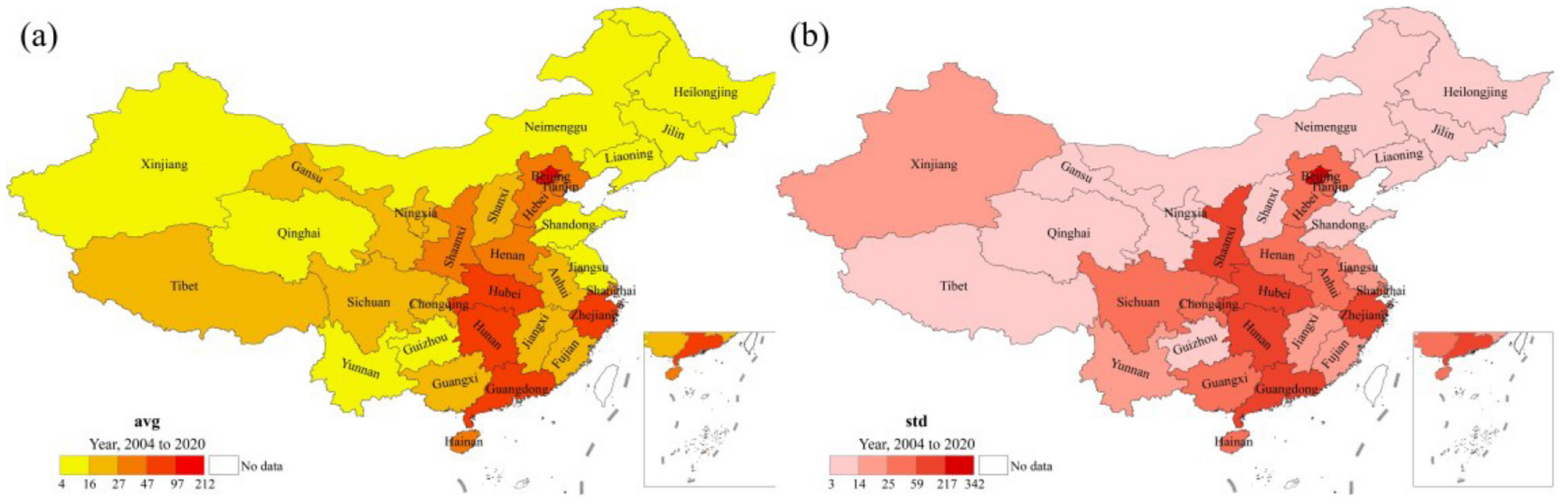
Spatial distribution of average **(a)** and standard deviation **(b)** of annual influenza incidence by province in mainland China, 2004–2020.

The average (avg) and standard deviation (std) of annual influenza incidence for each province in mainland China from 2004 to 2020 were calculated, and their spatial distribution is mapped (Figure 3). To highlight the spatial differences, the natural breaks method (53) was employed to classify the value range into five categories. From Figure 3a, it can be observed that the average number of cases per province varies significantly, with an overall average of 50 cases per 100,000 people. Beijing has the highest average incidence at 211.94 cases per 100,000 people, while Heilongjiang has the lowest at 4.19 cases per 100,000 people, representing a difference of more than 50 times. Provinces with higher averages are located in the southeastern region of China, while the northeastern and northwestern areas have relatively lower averages, overall showing a clustered pattern. The 10 provinces with significantly higher averages, in descending order, are Beijing, Zhejiang, Guangdong, Hunan, Hubei, Shaanxi, Hebei, Hainan, Henan, and Shanghai.

From Figure 3b, it can be observed that the spatial distribution of std is largely similar to that of avg, with a correlation coefficient of *r* = 0.96 (*p* < 0.001). The ten provinces with significantly higher standards are ranked as follows: Beijing, Zhejiang, Hunan, Guangdong, Shaanxi, Hubei, Sichuan, Hainan, Henan, and Chongqing. Among these, eight provinces share the same ranking as average. This indicates that avg is correlated with std, meaning that provinces with higher averages tend to experience greater fluctuations and are therefore less stable. In summary, provinces with higher average values undergo greater fluctuations and are consequently more unstable, and vice versa.

**Figure 4:**
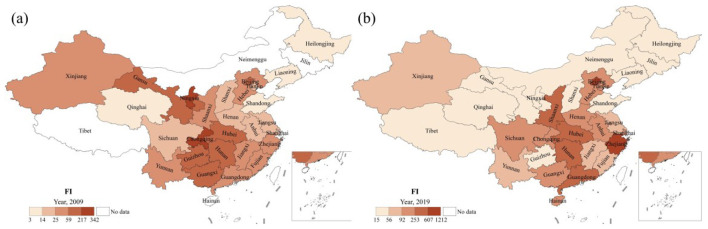
Spatial patterns of annual influenza incidence across provinces in mainland China for the years 2009 **(a)** and 2019 **(b)**.

As described in Figure 2, significant influenza outbreaks occurred in mainland China in both 2009 and 2019. Figure 4 illustrates the spatial distribution of annual influenza incidence across provinces in mainland China for those years. In 2009, the annual incidence was relatively high in the central-south region of China (Chongqing, Hubei, Hunan, Guizhou, Guangxi) and the central-western region (including Ningxia, Gansu, and Xinjiang). Compared to 2009, the annual incidence in 2019 was 14 times higher, although the overall spatial distribution patterns remained essentially consistent. The high-incidence provinces, ranked in descending order, are: Beijing, Zhejiang, Hunan, Guangdong, Shaanxi, Hubei, Sichuan, Henan, and Chongqing. This indicates that the annual influenza incidence exhibits significant heterogeneity, primarily concentrated in the central-southern and eastern regions of China. The comparison between these 2 years shows that influenza incidence varies significantly across regions and years, with outbreaks mainly occurring in the central and eastern parts of China.

The influenza incidence in 2009 and 2019 shows a spatial pattern similar to the average and standard deviation of annual influenza incidence during the 2004–2020 period (Figure 3). Notably, in 2019, the Pearson correlation coefficients, with their respective averages and standard deviations, were 0.94 (*p* < 0.001) and 0.99 (*p* < 0.001). This indicates that provinces with higher averages (avg) and standard deviations (std) are more susceptible to influenza outbreaks, and these provinces are mainly located in the southern and eastern regions of China. In other words, a larger baseline, combined with greater fluctuations, increases the likelihood that influenza incidence will exceed previous levels.

#### 3.2.2 Spatial pattern of change trends

**Figure 5:**
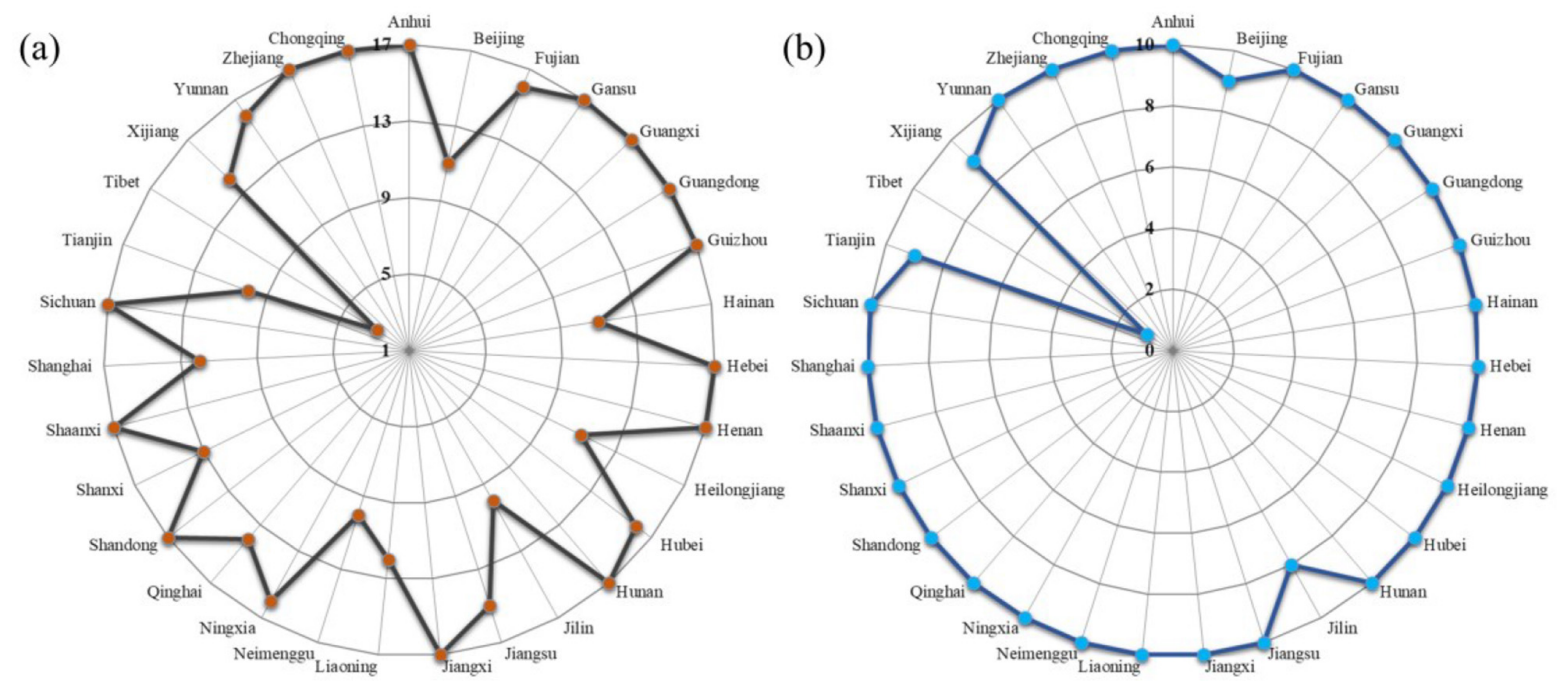
Number of years with complete influenza surveillance data for 31 provinces in mainland China, 2004-2020 **(a)** and 2011-2020 **(b)**.

The dataset used in this study spans a 17-year period from 2004 to 2020; however, not all provinces have complete records. Figure 5 shows the number of years with complete influenza surveillance data for 31 provinces in mainland China, from 2004 to 2020 and from 2011 to 2020. Although only 14 provinces have complete data records from 2004 to 2020, with Tibet having only 3 years of complete data, the data length of the remaining 30 provinces exceeds 9 years, meeting the requirements for trend modeling. Considering the differences in data lengths, we also analyzed the trend changes from 2011 to 2020. During this period, only the data from Beijing, Jilin, Tianjin, and Tibet are incomplete. Except for Tibet, which has only 1 year of complete data, the remaining 30 provinces have data lengths exceeding 8 years. Since Tibet has < 5 years of complete data records in both time periods, we excluded this region from subsequent trend analysis.

**Figure 6:**
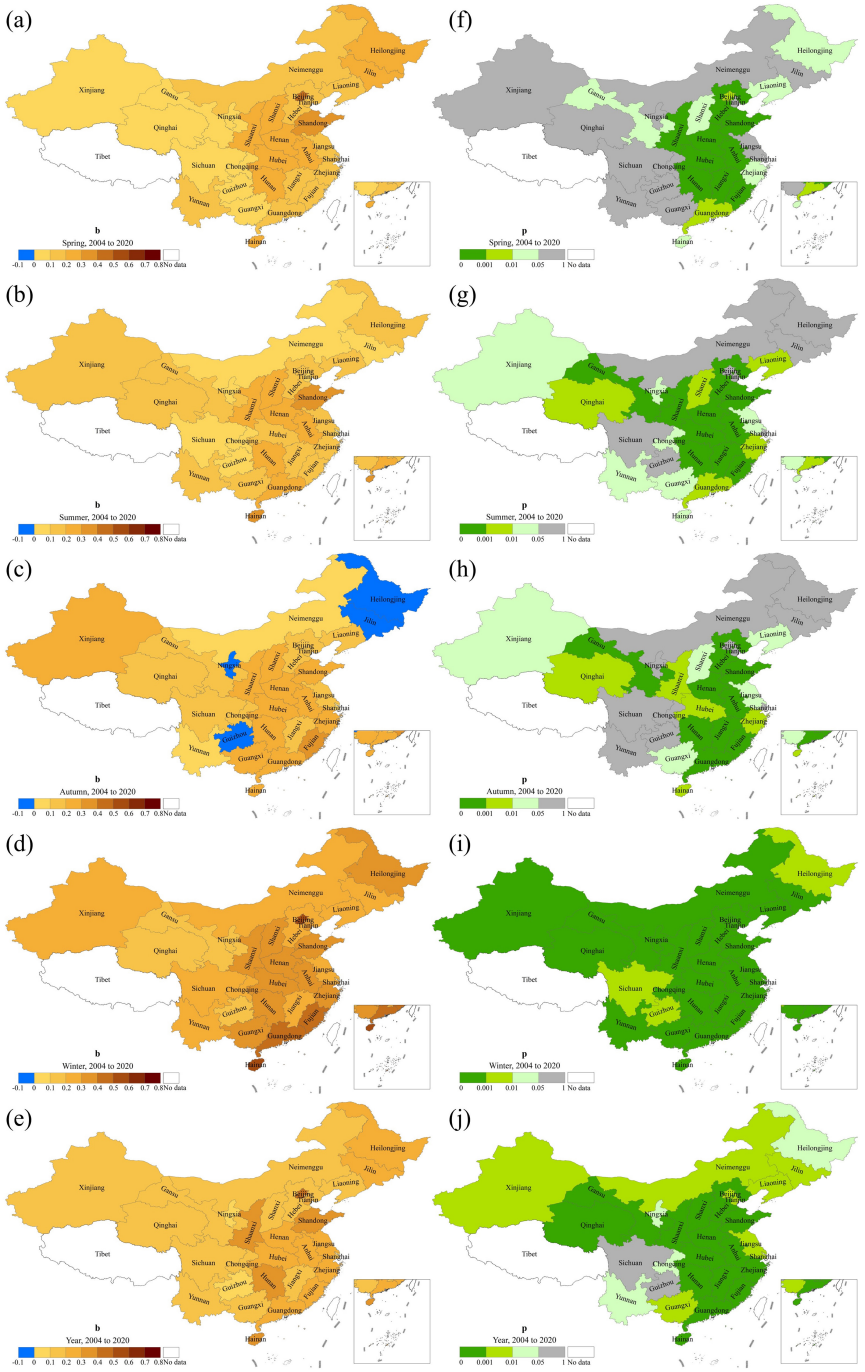
Spatial patterns of trend and significance of influenza incidence by season in mainland China, 2004–2020. *b* represents the change rate **(a–e)** and *p*-value is derived from the *F* test **(f–j)**.

Figure 6 illustrates the trend of influenza incidence in mainland China across different seasons from 2004 to 2020. It can be observed that influenza incidence across all four seasons exhibits an overall increasing trend, with a primary concentration in the southeastern and central-western regions. The average growth rate in spring is 0.2254/a, with 17 provinces passing the significance test (*p* < 0.05), mainly located in the southeastern region. The incidence of influenza in summer also shows an increasing trend. A total of 22 provinces exhibit a significant upward trend, with an average growth rate of 0.1872/a. Compared to spring, the provinces with significant increasing trends in summer have expanded to southern and western China. In autumn, the incidence of influenza in Guizhou, Heilongjiang, Jilin, and Ningxia exhibits a decreasing trend; however, this decrease is not statistically significant. The remaining 26 provinces show an increasing trend, among which 19 provinces passed the significance test, with an average growth rate of 0.2112/a. All 30 provinces exhibit a significant increase in winter, with an average growth rate of 0.3101/a.

It is worth noting that, except for three provinces showing a nonsignificant decreasing trend in autumn, most provinces show an increasing trend. From 2004 to 2020, a total of 15 provinces exhibited significant growth across all seasons, ranked in order of highest to lowest growth rate as follows: Gansu, Liaoning, Hebei, Shanxi, Jiangxi, Fujian, Henan, Guangdong, Anhui, Zhejiang, Hubei, Hunan, Shaanxi, Shandong, and Hainan. Their annual growth rates range from 0.1168/a to 0.3989/a. The annual incidence in mainland China shows an increasing trend, with an average growth rate of 0.2303/a. Except for Guizhou and Sichuan, the growth trends of the remaining 28 provinces passed the significance test.

**Figure 7:**
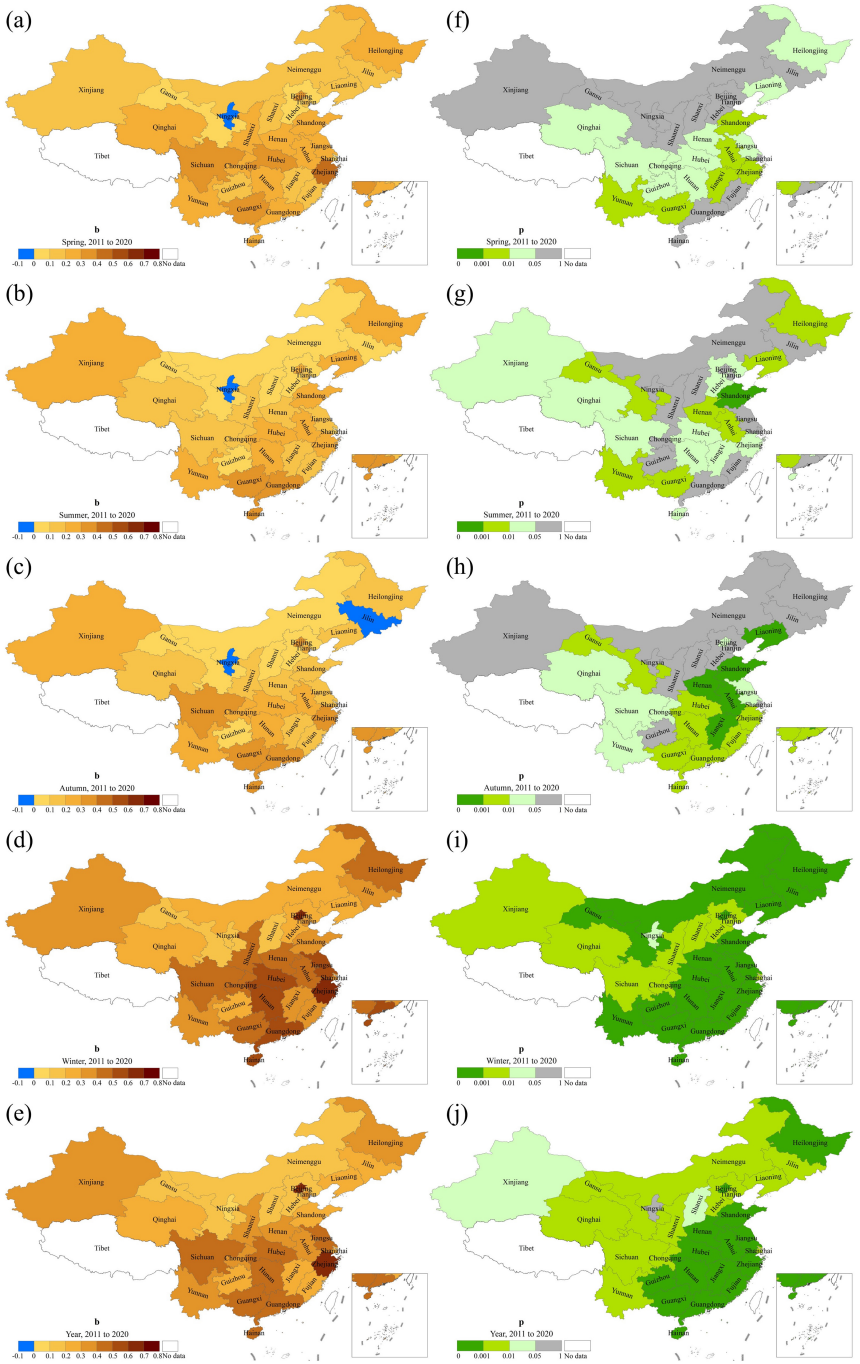
Spatial patterns of trend and significance of influenza incidence by season in mainland China, 2011–2020. *b* represents the rate of change **(a–e)** and *p*-value is derived from the F test **(f–j)**.

Figure 7 shows the trend of influenza incidence in mainland China for each season from 2011 to 2020. In spring, all provinces except Ningxia exhibited an increasing trend, with 16 provinces passing the significance test. The average growth rate during this period is 0.2586/a. Influenza incidence in summer also shows an increasing trend in all provinces except Ningxia, with 17 provinces passing the significance test and an average growth rate of 0.2197/a. In autumn, except for Jilin and Ningxia, influenza incidence increases in the remaining 26 provinces. Of these, 19 provinces pass the significance test, with an average growth rate of 0.2341/a. Similar to the period 2004–2020, during 2011–2020, influenza incidence in winter across 30 provinces in mainland China shows a significant increasing trend, with an average growth rate of 0.3887/a. The annual incidence of influenza increases across all 30 provinces. Except for Ningxia, the trend in the other 29 provinces passes the significance test, with an average growth rate of 0.3235/a. Overall, influenza incidence increased from 2011 to 2020, primarily in southeastern and western provinces, with the most significant growth occurring during winter. Twelve provinces showed significant increasing trends across all four seasons, ranked by growth rate from highest to lowest: Zhejiang, Hubei, Sichuan, Hunan, Guangxi, Anhui, Yunnan, Henan, Shandong, Qinghai, Liaoning, and Jiangxi. The annual incidence growth rates ranged from 0.2318/a to 0.6008/a.

Through a comparative analysis of Figures 6, 7, it can be seen that the influenza incidence in mainland China exhibits an overall increasing trend from 2004 to 2020 and from 2011 to 2020. Although some provinces exhibit a decreasing trend in certain seasons, the trend is not significant. Since the incidence in winter accounts for more than half of the annual total, and all provinces show a significant increasing trend in winter, the spatial patterns of both periods are basically consistent. Although the number of provinces with significant trends from 2011 to 2020 has decreased compared to 2004–2020, the average growth rate is higher.

**Figure 8:**
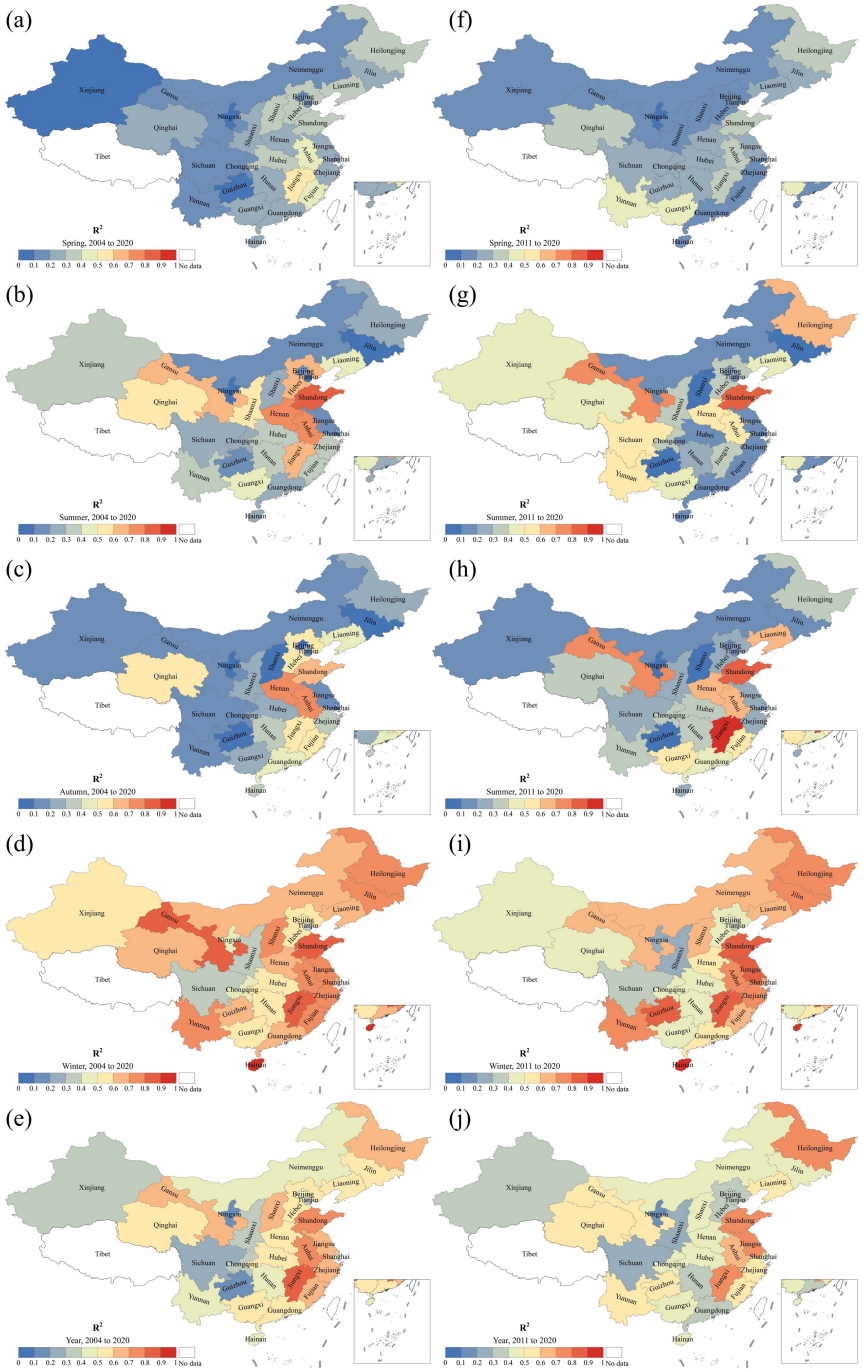
Goodness of trend model fitting of influenza incidence change in mainland China during 2004–2020 **(a–e)** and 2011–2020 **(f–j)**.

Figure 8 illustrates the Goodness of fit for the trend model of influenza incidence changes in mainland China from 2004 to 2020 and from 2011 to 2020, represented by R^2^. The left column shows the R^2^ values of the trend model for seasonal incidence changes from 2004 to 2020, while the right column presents those from 2011 to 2020. It can be observed that the average R^2^ values over 2004 to 2020, in descending order, are: winter (0.64) > summer (0.36) > autumn (0.30) > spring (0.25). The average *R*^2^ of the trend models for annual incidence across 30 provinces in mainland China is ~0.5. The top 10 provinces with the best-fitting results, in descending order, are: Hainan > Shandong > Beijing > Hunan > Shaanxi > Fujian > Anhui > Guangdong > Shanxi > Hubei. The average *R*^2^ from 2011 to 2020, in descending order, are: Winter (0.60) > Autumn (0.35) > Summer (0.32) > Spring (0.23). The *R*^2^ for the annual incidence across the 30 provinces in mainland China is 0.48. Provinces with an average *R*^2^ > 0.5 over the four seasons, in descending order, include: Shandong > Jiangxi > Gansu > Anhui > Henan > Heilongjiang > Liaoning > Yunnan > Guangxi > Hainan. The R^2^ values for the two periods, 2011–2020 and 2004–2020, show both similarities and differences. The similarity is that the best fit occurs in winter and the worst in spring in both periods. Additionally, provinces with higher *R*^2^ are mainly concentrated in the southeastern, central-western, and northeastern regions of China. Differences are evident in the varying value ranges and distributions of *R*^2^ within the same season.

### 3.3 Associations between the characteristics of influenza incidence

To explore the associations between characteristics, we examined the correlations among the annual change rates (*b*), average (avg), and standard deviation (std) of influenza incidence across 30 provinces in mainland China during the periods of 2004–2020 and 2011–2020 (Table 2). There are significant positive correlations among these three variables, except for non-significant correlations between *b* vs. avg, and *b* vs. std during autumn in 2004–2020. Avg and std are significantly positively correlated across all seasons during both periods, with *r* ≥ 0.75 (*p* < 0.001). This suggests that provinces with higher influenza incidence experience more intense fluctuations and greater variability. Overall, b is positively correlated with avg, with *r* ≥ 0.37, indicating that provinces with higher incidence tend to have greater growth. The correlation between *b* and std is stronger than that between *b* and avg, with *r* ≥ 0.43. The volatility of influenza incidence is linked with trends, indicating that greater volatility corresponds to larger interannual variability.

**Table 2:** Correlations between change rate (b), average (avg) and standard deviation (std) of annual influenza incidence in 30 provinces of mainland China.

**Time period**	**Season**	**Pearson correlation coefficient (** ***r*** **)**		
		**avg vs. sth**	**b vs. avg**	**b vs. std**
2004–2020	Spring	0.98^***^	0.56^***^	0.60^***^
	Summer	0.96^***^	0.37^*^	0.43^*^
	Autumn	0.77^***^	0.22	0.12
	Winter	0.96^***^	0.61^***^	0.61^***^
	Year	0.97^***^	0.59^***^	0.63^***^
2011–2020	Spring	0.97^***^	0.50^**^	0.58^***^
	Summer	0.95^***^	0.47^**^	0.56^***^
	Autumn	0.75^***^	0.47^**^	0.63^***^
	Winter	0.98^***^	0.69^***^	0.75^***^
	Year	0.98^***^	0.72^***^	0.80^***^

^*^p ≤ 0.05, ^**^p ≤ 0.01, ^***^p ≤ 0.001. Non-significant results are not marked. avg, std and b are the average, standard deviation and change rate, respectively.

**Figure 9:**
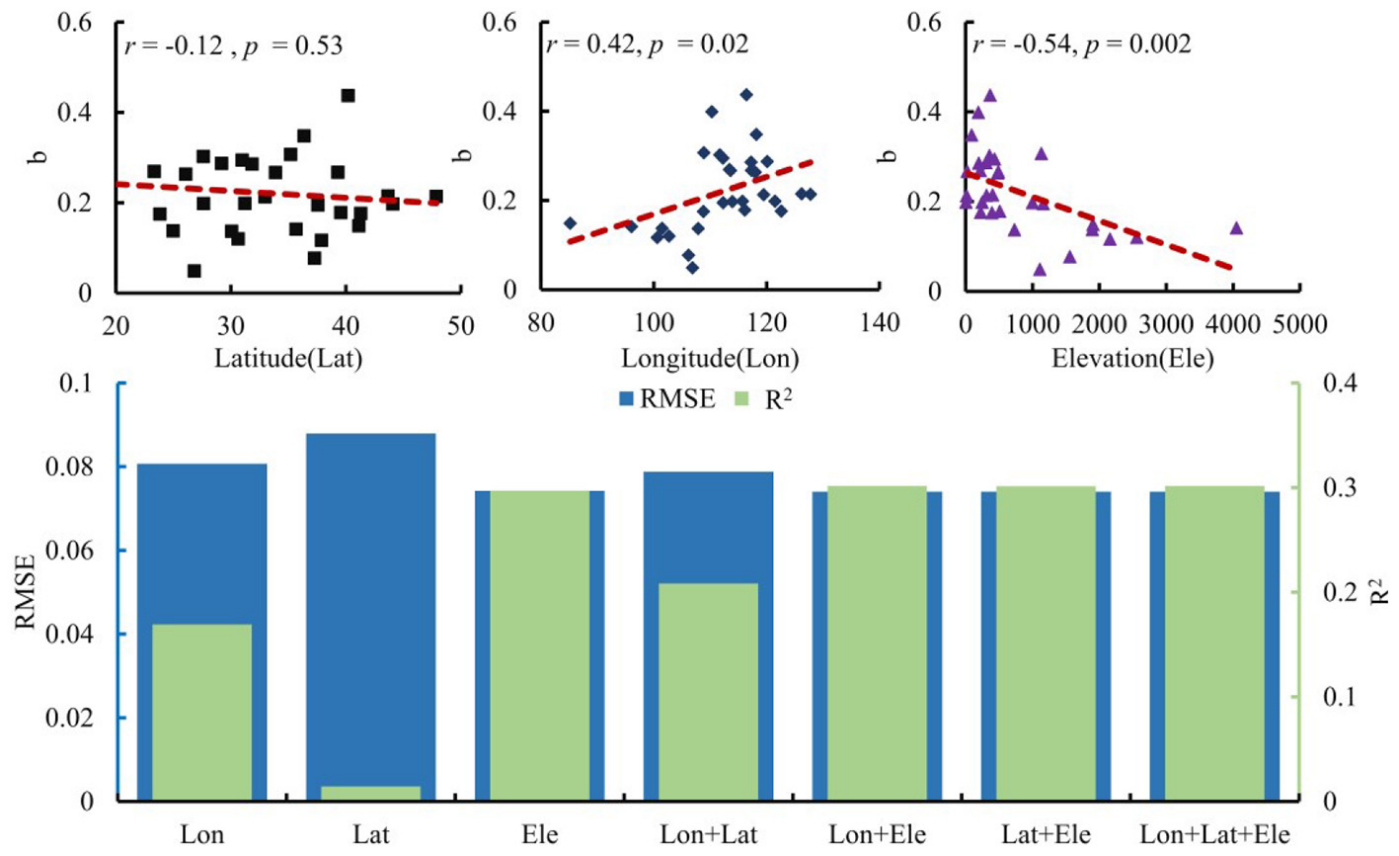
Association between the change rate of annual influenza incidence in 30 provinces of mainland China from 2004 to 2020 and geographic factors.

Furthermore, we also analyzed the spatial dependence of *b* by employing a regression model with longitude (Lon), latitude (Lat), and elevation (Ele). As shown in Figure 9, there is a positive correlation between *b* and Lon, while a negative correlation exists between *b* and Ele, with Ele having a stronger explanatory power for *b* than Lon. The change rate in the annual incidence exhibited differentiation based on elevation and longitude. High *b* values are primarily concentrated in provinces near 115°E and at elevations below 500 m. According to the combined ranking based on *R*^2^ (from high to low) and RMSE (from low to high), the explanatory power of these geographical variables is as follows: Lon + Lat + Ele = Lon + Ele > Lat + Ele > Ele > Lon + Lat > Lon > Lat. Longitude and elevation have the strongest explanatory power for the spatial distribution of *b*, which is equal to the total explanatory power of all three geographical variables. The distribution pattern of *b* across latitudes is not obvious. However, the explanatory power of latitude and elevation for *b* is stronger than that of elevation alone. Given that their combined explanatory power is significantly stronger than each of their individual explanatory powers, and is not a simple summation of their individual contributions, it can be concluded that the spatial distribution of *b* exhibits local clustering, with elevation playing a non-negligible role.

## 4 Discussion

Influenza presents a significant public health challenge in China each year, with an average annual incidence rate of 45 cases per 100,000 people, leading to a considerable disease burden. For example, in 2019, influenza-related national economic losses amounted to 26.38 billion yuan (42). Analyzing the spatiotemporal patterns of influenza incidence both qualitatively and quantitatively is essential for effective epidemic monitoring, prevention, and control. Since most previous studies focused on influenza incidence within specific regions or timeframes, making it hard to fully understand the spatiotemporal patterns across mainland China, we conducted this multi-province, multi-scale analysis. This study thoroughly examined the characteristics and trends of influenza incidence in China from 2004 to 2020 by consolidating all available provincial-level surveillance data. We identified significant variations in incidence across different years and provinces. These results align with inter-country analyses and provide evidence of the heterogeneity of influenza activity within a country (33). This highlights the importance of developing region-specific prevention and control strategies.

Revealing the seasonal characteristics of influenza incidence change is crucial for policymakers to determine the optimal timing for influenza vaccination campaigns (31). We found that the monthly incidence of influenza in mainland China follows a U-shaped curve throughout the year. In other words, the peaks of influenza incidence typically occur in January and December, while the lowest point is in June. This aligns with previous studies conducted in specific regions or at different periods (10, 19, 34, 45, 48, 54). This suggests that the seasonality of influenza incidence is closely tied to climate and environmental factors (36, 52). However, the high incidence rate in March is noteworthy and may relate to sudden temperature changes (9). After March, influenza incidence drops significantly, with only sporadic outbreaks in some provinces. These findings are essential for developing targeted prevention and control measures tailored to local conditions (38). For example, influenza vaccination is the most effective way to prevent infection and reduce severe influenza-related complications (45), especially in the older adults and young children (54). Public health officials can focus limited medical resources in key locations and at critical times to respond effectively to potential outbreaks.

The influenza prevention and control situation in mainland China remains severe. From 2004 to 2020, the influenza incidence in mainland China showed significant increases on a monthly, seasonal, and annual basis, with a particularly notable trend after 2010. These increasing trends are primarily reflected in the growth during winter months, especially in the southern and eastern regions. These trends align with previous related studies (33, 55, 56). Moreover, we found that when significant increases are observed in spring, summer, and autumn, the winter influenza incidence and the annual total incidence also show a significant upward trend, particularly in the southeastern regions. Several factors may explain the rising trend of influenza during this period. First, increased efforts in influenza surveillance and the adoption of new technologies may be the primary reasons for the rise in influenza cases (48). The influenza surveillance network in China has gradually expanded and improved since 2009, now covering all prefectural and municipal hospitals, a few county hospitals and centers for disease control and prevention in 31 provinces (54). As more sentinel hospitals implement electronic health information systems, the quantity and quality of data collection have improved (10). The use of new technologies such as molecular diagnostic techniques, point-of-care testing, and antigen testing has enhanced the detection efficiency and diagnostic accuracy of influenza cases. With the continual development of the surveillance network and advancements in diagnostic technologies, more influenza cases are being identified and reported promptly. Second, the government and healthcare institutions have strengthened education and awareness campaigns on infectious diseases, such as influenza, through various channels (e.g., television, the internet, and community activities), thereby increasing public awareness of disease prevention and health management. During this period, a major influenza pandemic occurred, and the public gained a deeper understanding of the dangers of influenza and the importance of preventive measures. The increased coverage of healthcare insurance in both urban and rural areas in recent years in China may also encourage people to utilize more healthcare services. This led to more people seeking medical attention proactively, increasing in reported cases. Furthermore, ongoing antigenic changes in influenza viruses and waning population immunity likely contribute to recurring susceptibility, providing an additional driver of rising reported incidence alongside improved surveillance coverage and diagnostics. During the study period, the rapid growth of air travel and high-speed rail transport in China facilitated the spread of influenza over larger areas and in shorter time frames (57). The combined effects of urbanization and climate change have made certain populations (such as low-income groups, the older adults, and children) more vulnerable, increasing the risk of influenza incidence.

This study clearly shows that the incidence of influenza forms a localized clustering pattern, mainly concentrated in developed provinces, where the growth rates are also the highest. This phenomenon may be linked to environmental conditions and levels of socioeconomic development (52), such as population density, age structure, attractiveness to both domestic and international travelers, and medical service capacity (10, 35, 58). This highlights the need for the development of effective local influenza planning strategies. The regional clustering of influenza also increases the risk of case spillover. If high-incidence areas can be contained locally, especially in densely populated and well-connected regions, it could benefit neighboring areas (43). Therefore, increasing surveillance in adjacent regions will help prevent and control local influenza outbreaks.

A better understanding of influenza incidence is crucial for optimizing surveillance networks and creating accurate forecasting models (26). We also provided evidence showing that the characteristics of influenza incidence in mainland China display spatial dependence. The growth rate of annual influenza incidence is positively correlated with longitude, multi-year average, and standard deviation, while it is negatively correlated with elevation. This indicates that the central-southern and eastern coastal regions of China, which are mostly economically developed, have good transportation and dense populations. Incorporating these factors into a spatial forecasting model could improve its accuracy. The link between the growth rate and latitude is not significant, likely due to the coarse spatial resolution of the data. Given China's vast size and diverse climates, it is expected that growth rates vary across latitudes. As influenza high-incidence zones exhibit more pronounced growth trends, regional disparities in influenza incidence are likely to increase. Variations in annual influenza incidence can also partly arise from differences in the baseline population susceptible to infection. This study lays the groundwork for future research on social and environmental influences. These disparities should be examined further as more data become available.

A large-scale, long-term analysis of influenza surveillance data can provide valuable information and a scientific basis for predicting influenza activity (8). We observed a significant positive correlation among the mean, variance, and rate of change. Accordingly, provinces with high influenza incidence tend to experience greater fluctuations and faster growth. Moreover, their spatial distributions were largely similar, such as high values clustered in localized regions of southern and eastern China. This finding has critical epidemiological implications for understanding influenza incidence dynamics in mainland China and developing effective prevention and control strategies. It reflects the interconnected multiple influences. Firstly, higher influenza incidence offers a larger host base for the virus's transmission, increasing opportunities for spread and leading to wider dissemination and regional diffusion. Secondly, fluctuations may be related to the combined effects of factors such as population immunity, viral mutations, and climate change. In provinces with a higher incidence, these factors may cause greater volatility. Finally, intense volatility can break through the current state and initiate a new phase of growth. As a result, provinces with greater volatility tend to show more pronounced growth trends. This also highlights the inherent cumulative effect of influenza incidence, where higher initial levels can lead to faster subsequent growth. This underscores the importance of enhancing surveillance and early warning systems in high-risk provinces, along with timely adjustments to prevention and control strategies to address potential outbreaks. From a public health management perspective, this correlation has significant early warning value. When a notable rise in incidence occurs in areas with high influenza activity, it is crucial to monitor neighboring regions and remain vigilant for possible subsequent outbreaks. This requires health authorities to implement more proactive and assertive control measures, such as expanding surveillance, increasing medical resource reserves, and strengthening public health interventions. Beyond vaccination as the primary prevention method, timely antivirals and calibrated non-pharmaceutical interventions (e.g., improved ventilation, masking in high-risk settings, reducing crowding, and stay-at-home advice for symptomatic individuals) serve as additional measures during epidemic and pandemic phases. Their targeted use, guided by surveillance thresholds and local healthcare capacity, can reduce peak burdens and protect high-risk groups while maintaining essential services. Incorporating these strategies into preparedness plans is especially relevant for provinces with rapid growth and high incidence volatility. Furthermore, this research offers new insights for optimizing influenza forecasting models. Current models do not fully consider the relationships among incidence levels, volatility, and trends, which could enhance prediction accuracy and efficiency. Future research will further investigate the specific mechanisms underlying this correlation, as well as the relative importance of various influencing factors, thereby providing a stronger scientific basis for influenza prevention and control.

Preventing and controlling influenza has been one of the biggest challenges to global public health. The incidence of influenza in mainland China has shown an increasing trend, making it a major public health concern. Influenza activity is often linked to excess mortality (42). As a lower-middle-income country with vast geographic area and a large population, China faces a significant disease burden associated with these outbreaks (9, 19). Influenza prevention and control remain major challenges in public health. Our analysis not only reveals the intra-annual variation in influenza incidence in mainland China and the increasing trends at monthly, seasonal, and yearly levels, but also examines the distribution patterns of these trends, offering new insights at the provincial level. The detailed understanding of the temporal and spatial patterns of influenza incidence in China helps us grasp the dynamics and regularities, which have important implications for macro-level prevention and control strategies (6). Additionally, considering China's key role in the global spread of influenza (43), its spatiotemporal patterns provide valuable information for other countries and regions.

## 5 Limitations

Despite the important findings of this study, some limitations remain. First, the results depend heavily on the quality of influenza surveillance data. There is a notable gap between the reported influenza incidence and the actual incidence (10, 38, 54, 59). This discrepancy arises because influenza incidence is based on patient data reported by the influenza surveillance network, which inevitably misses infections that resolve on their own or are self-treated. As the scope of influenza surveillance expands and surveillance protocols are continually improved (55), the completeness and consistency of the dataset may vary, introducing an unknown level of bias (41, 60). Second, the analysis was performed using provincial administrative units, resulting in a coarse spatial resolution. Significant differences also occur within provinces. Finer granularity in the time series would reveal more specific spatiotemporal patterns and support urban-rural classification analysis, avoiding over-representation of provinces with low population density. Analyzing at finer spatial scales (prefecture/county, urban–rural strata) is necessary to address within-province heterogeneity and local clustering. When finer-grained data become available, future work will utilize multilevel and spatial dependence models to better understand hotspot–spillover structures. Finally, due to variations in climate, healthcare access, socioeconomic factors, and urban-rural disparities, conclusions drawn from provincial influenza surveillance data need further validation for broader application at the city and county levels. As more detailed subdivided and classified data become available in the future, more precise patterns may be identified.

We will address these limitations in future studies. The heterogeneity analysis will include a wider range of variables related to population, society, climate, and the environment to better characterize the spatiotemporal patterns. More detailed influenza surveillance data will be collected to understand the features and differences among various populations at the community level. Additionally, the collective epidemiology of all circulating influenza viruses cannot distinguish the contributions from individual subtypes. Variations in transmissibility, severity, or vaccine effectiveness between subtypes may influence the observed aggregate trends (61). The aggregated influenza incidence dataset from the national notifiable disease reporting system lacks laboratory-typed influenza subtypes or B lineages, which limits subtype- or lineage-specific spatiotemporal analyses and restricts conclusions about viral evolution. Future research will incorporate virological data from the national influenza surveillance network to better understand subtype/lineage-specific dynamics and improve forecasting accuracy. The current study did not include hospitalization or mortality data, such as data on severe acute respiratory infections (SARI), which limits the ability to assess clinical severity and additional disease burden. Future research will link incidence data with hospitalization and mortality registries to understand disease severity gradients and estimate the excess disease burden.

## 6 Conclusions

Since 2000, China has undergone unprecedented socio-economic development, with significant progress made in controlling infectious diseases. However, influenza, a common and environmentally influenced acute respiratory infection, remains a significant public health challenge. Its incidence surged dramatically by 20 times between 2004 and 2020, emphasizing the ongoing severity of the problem. This study's findings enhance understanding of influenza dynamics in China by clarifying its characteristics, trends, and patterns of change. They offer a scientific foundation for public health authorities to develop targeted strategies for influenza prevention, control, and management. Authorities should improve dynamic surveillance and information sharing on influenza activity, optimize medical resource allocation, and strengthen forecasting and rapid response efforts.

## Data Availability

Publicly available datasets were analyzed in this study. This data can be found here: https://www.phsciencedata.cn.
